# Protective Effects and Mechanisms of Rosuvastatin on Acute Kidney Injury Induced by Contrast Media in Rats

**DOI:** 10.1155/2020/3490641

**Published:** 2020-05-18

**Authors:** Zehui Jiang, Jun Zhang, Yuanan Lu

**Affiliations:** ^1^Jiangxi University of Traditional Chinese Medicine, Nanchang 330006, China; ^2^Department of Cardiovascular Medicine, The 908th Hospital of Chinese People's Liberation Army Joint Logistic Support Force, Nanchang 330006, China; ^3^Department of Public Health, University of Hawaii at Manoa, Honolulu, HI 96822, USA

## Abstract

**Objective:**

To explore the protective effect and mechanism of rosuvastatin on acute renal injury induced by a nonionic hypotonic contrast medium in rats.

**Methods:**

Forty-eight healthy adult SD rats were randomly divided into three groups: normal control group (NC); contrast medium control group (CM); and rosuvastatin intervention group (RI). The RI group was intragastrically administered with a 10 mg/kg of rosuvastatin 12 h prior to the contrast exposure. All rats in CM and RI groups were inoculated with 10 mL/kg of chemical (IV) while the same volume of saline for the NC group. At 24 h and 72 h posttreatments, pathomorphological changes of renal tubules were documented, respectively, and several biochemical indicators were tested to assess renal injury of experimental rats.

**Results:**

Compared with the CM group, rats in the RI group showed significantly reduced injury of kidneys and decreased levels of biochemical indicators such as blood Scr, blood Cys-C, urine NAG, urine *α*1-MG, and urine mALB. The serum Hs-CRP in the CM group increased significantly from 24 h to 72 h (*p* < 0.05), but this was not observed in the rats of the RI group. In addition, SOD activity in the RI group was significantly increased (*p* < 0.01) while SOD activity in renal tissue decreased significantly with time in the CM group (*p* < 0.05).

**Conclusion:**

Short-term intervention with rosuvastatin can lead to reduced kidney damage associated with the contrast agent by reducing the levels of inflammatory factors and oxidative stress. Thus, rosuvastatin intervention has a protective effect on rats from contrast-induced nephropathy.

## 1. Introduction

Contrast-induced nephropathy (CIN) is one of the main causes of acute kidney injury (AKI), which has increased in recent years due to the development of modern imaging radiology techniques and interventional methods and increased use of contrast agents [1, 2]. In addition of having a poor prognosis, CIN patients are often encountered with increased incidence of cardiovascular disease, all-cause mortality, and end-stage renal disease, and prolonged time for hospital stay [3]. The incidence of CIN is heterogeneous with a wide range from 1.3% to 37.7% [4]. The occurrence of CIN is known to be affected by a few factors including the patient's underlying disease status, research findings, intervention approaches, and preventive measures [4]. The incidence of CIN after coronary angiography (CAG) is 10% to 15%, and the incidence of CIN can be as high as 50% in high-risk groups with multiple CIN risk factors [5].

Several methods are presently available for clinically treating CIN, including postcontrast hydration therapy and alkalized urine, changing the type of contrast agent, calcium channel blockers, and vasodilators. Clinical-related studies have recently revealed that vastatin drugs have a certain effect on the prevention of CIN [6]. In this paper, experimental studies were conducted to further analyze the possible protection and related mechanism of rosuvastatin on acute kidney injury induced by contrast medium in rats. These new findings may provide the necessary theoretical basis for clinical research applications.

## 2. Materials and Methods

### 2.1. Experimental Animals

Forty-eight healthy adult male SD rats were used in this study, and their body weight ranged from 250 to 350 g. These rats were randomly divided into three groups: normal control group (NC); contrast-medium control group (CM); and rosuvastatin intervention group (RI). There were 16 animals per group, and the basic information such as the age and weight of the rats in each of three groups are relatively small, with no statistical significance (*p* > 0.05).

### 2.2. Experimental Chemicals

Rosuvastatin calcium tablets were purchased from AstraZeneca Pharmaceuticals Co., Ltd.(China), and Iopromide 370 was from Bayer Pharmaceuticals with an iodine content of 370 mg/mL (50 mL/bottle).

### 2.3. Experimental Methods

#### 2.3.1. Subgroups

16 rats in each group were equally divided into two subgroups at random. These two subgroups, each consisting of 8 animals, were sacrificed at 24- and 72-hour posttreatment (PT), respectively, and tested for rosuvastatin-mediated protective effect on acute renal injury induced by a nonionic hypotonic contrast agent (Iopromide 370).

#### 2.3.2. Preparation of Rosuvastatin Suspension

Rosuvastatin calcium tablets were ground with a sterile mortar/pestle and then dissolved in 0.9% NaCl solution. Completely dissolved suspension was stored in 4°C until use.

#### 2.3.3. Treatment Procedures

Experimental rats in the three groups were treated as follows: (i) CM group: 16 rats were intragastrically fed with 2 mL of 0.9 NaCl solution. After 12 hours, each of these animals received a single dose of tail vein (tv) injection of Iopromide 370 (10 mL/kg body weight (bw)). These animals were fed intragastrically with 2 mL of 0.9% NaCl solution daily and examined for the renal injury at the selected time points, (ii) RI group: experimental rats in were intragastrically administered with a rosuvastatin suspension at a dose of 10 mg/kg bw. After 12 hours, all 16 animals received a single dose of the tv injection of Iopromide 370 (10 mL/kg bw). These animals were continuously fed with 10 mL/kg bw rosuvastatin suspension every day and examined for potential protection of rosuvastatin on renal injury at the selected times. (iii) NC group: 16 rats in this group received the same treatment as the CM group except for the single dose of tv injection of 0.9% NaCl at 10 mL/kg bw. These animals were fed with 2 mL of 0.9% NaCl daily and used as a negative control of the test.

At two time points (24 h and 72 h) after angiography, 8 rats from each group were anesthetized, respectively, with an intraperitoneal injection of 10% chloral hydrate at 3 mL/kg bw. Rat blood was drawn from the heart with blood vessels for the detection of Scr, Cys-C, and Hs-CRP. The kidneys of experimental rats were identified following a U-shaped abdomen incision, and tissue samples were harvested with a sharp surgical blade and fixed immediately by putting them in a 10% formaldehyde aqueous solution.

### 2.4. Observation Indicators

#### 2.4.1. Histopathology

Serials sections (3 *µ*M) were prepared from paraffin-embedded renal tissues of experimental rats, stained with hematoxylin and eosin (HE), and examined for pathomorphological changes of renal tubules with a light microscope at 400x magnification (Olympus Ix53, Japan). The renal tubular injury was scored according to the method described previously [7]. In brief, a total of 10 fields of external medulla were captured from each specimen and observed from the upper left and right, lower left and right, and middle in sequence. Renal tubular injury was scored at five levels basing on tissue damages: 0 = normal with no apparent injury; 1 = minor injury (≤5%); 2 = mild injury (≤25%); 3 = moderate injury (≤75%); and 4 = severe injury (>75%).

#### 2.4.2. Biochemical Indicators

The experimental rats from the three groups were similarly processed and tested for the following biology indicators at 24 h and 72 h after angiography, respectively: rat serum cystatin C (Cys-C), serum creatinine (Scr), urine *α*1-microglobulin (*α*1- MG), urine microalbumin (mALB), urine N-acetyl-BD-glucosidase (NAG) levels, serum high-sensitivity C-reactive protein (Hs-CRP), and superoxide dismutase (SOD) activity in kidney tissues.

### 2.5. Statistical Analysis

All experimental results were expressed by *x* ± *s* (means ± SD). The homogeneity test of variance was performed using the system statistical software SPSS Statistics 17, and the comparison between groups was performed by using the SNK test. The comparison within the group was performed by the *t*-test, *α* = 0.05. *p* < 0.05 is considered to be significantly different.

## 3. Results

### 3.1. General Observation

Rats in three groups were observed daily after the treatment and comparatively recorded: rats in the NC group were in good mental condition, active, white and smooth hair color, normal eating and drinking water, urine output, and urine color were normal. Rats in the CM group showed poor response sensitivity, less movement, and dull yellowing, with normal drinking water on the first day, followed by less drinking and less intake of food, and urine output began to decrease, but urine color was clear, no obvious turbidity and hematuria. The observed results for the rats in the RI group were between NC and CM groups.

### 3.2. Morphological Changes of Renal Tubular Cells

**Figure 1:**
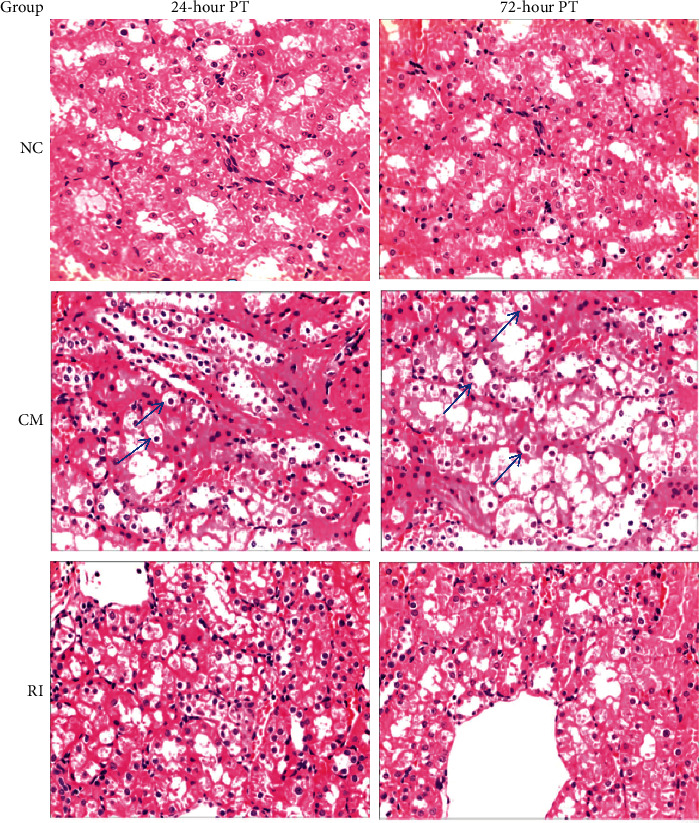
Morphological and pathological changes of renal tubular cells occurred in rats of three experimental groups, showing the effect of rosuvastatin on reduced apoptosis of renal tubular cells in the RI group (arrows showing epithelial cells with vacuolar-like degeneration). HE staining, magnification 400x.

Experimental rats were comparatively examined for morphological alteration of renal cells at 24 h and 72 h after tv injection of the contrast medium. As shown in Figure 1, the renal tubular cells in the NC group appeared flat and were neatly arranged with no swelling of the cytoplasm. No apparent change was detected between two time points. The epithelial cells in the CM group appeared swollen; the cavity is narrow and irregular, the epithelial cells are obviously vacuolar-like degeneration, and some renal tubular cells are necrotic and shed. Compared to 24 h observation, no further change in morphology was visualized in renal tubular cells at 72 h in this group. In the RI group, similar changes as detected in the CM group were documented, but the cellular damage was much less (Figure 1). The morphological changes between the two time points were not significant between the two subgroups.

Analysis of renal tubular injury in rats of different groups showed that the renal tubular damage score in CM and RI groups is significantly higher than the scores detected in the NC group (*p* < 0.05) (Table 1). Compared to the CM group, the rats in the RI group had a statistically significant lower score (*p* < 0.05). As shown in Table 1, there was no significant difference in the renal tubular injury between 24 h and 72 h in each group (*p* > 0.05).

### 3.3. Changes in Serum Biochemical Indicators

#### 3.3.1. Levels of Serum Scr, Blood Cys-C, Urine NAG, Urine *α*1-MG, Urine mALB, and NAG in Experimental Rats

The blood Scr, blood Cys-C, urine NAG, urine *α*1-MG, and urine mALB in the CM and RV groups were all significantly increased as compared to the NC group.

However, the deterioration of the above indicators was significantly lower in the RI group than in the CM group (Table 2). Compared with 24 h after Iopromide 370 injection, renal function in the CM group was further worsened at 72 h, while renal function damage in the RI group was not intensified, and the NAG index even showed a slight improved in the 72 hr RI subgroup (Table 3).

#### 3.3.2. Detection of Hs-CRP in Experimental Rats

As shown in Table 4, the level of Hs-CRP in both CM and RI groups increased as compared to the NC group. However, the change in the RI group was significantly lower than that detected in the CM group (*p* < 0.05). Hs-CRP in the CM group was further increased in the 72 h subgroup compared with the 24 h subgroup. In contrast, the Hs-CRP in the RI group showed a slight decrease with time (Table 4).

#### 3.3.3. SOD Changes in Experimental Rats

The test results of SOD for the three groups are summarized in Table 5. Compared with the NS group, the SOD level in the CM and RI groups was significantly reduced (*p* < 0.01). Compared to the CM group, the reduction in the RI group was even significantly lower (*p* < 0.01). Compared to the two time points, SOD detected in the CM group was significantly reduced in the 72 h subgroup than that in the 24 h subgroup. However, protective SOD in the RI group increased slightly in the 72 h subgroup as compared to the 24 h subgroup (Table 5).

## 4. Discussion

Present studies have revealed that the occurrence of CIN is closely related to renal vasoconstriction and subsequent renal medullary ischemia and hypoxia, renal tubular obstruction, apoptosis, inflammatory response, and oxidative stress [8]. However, the exact pathogenesis of CIN is still unclear. With the gradual deepening of clinical application and research, statins have shown important multiple functions in recent years, including its anti-inflammatory, antioxidant, antithrombotic, and antiproliferative effects [9]. This study was designed to clarify the contrast-induced renal injury in rats and understand possibly the protective effect of rosuvastatin, which allows us to explore the preventive mechanism of rosuvastatin on contrast nephropathy from anti-inflammatory and antioxidative stress.

The basic clinical pathological changes of contrast nephropathy include acute tubular necrosis, severe granule and vacuole degeneration of renal tubular epithelial cells, and then disintegration and shedding. In addition, collective duct lesions are particularly serious, with diffuse edema in the renal interstitium and no obvious glomerular lesions [10]. In this study, HE staining was used to stain kidney tissue sections and pathology, and morphological changes were observed in the CM group including swollen epithelial cells in the renal tubules with vacuole-like degeneration and necrotic renal tubular cells. Similar changes were also observed in the RI group but with less cellular damage, indicating that short-term intervention with rosuvastatin played a certain protective role.

The current clinical diagnosis of CIN depends on changes in Scr levels, but Scr is not sensitive to early renal damage [11]. Cys-C is a small-molecule protein, which is positively charged. Because of a greater molecular weight over creatinine, Cys-C is easier to reflect early changes in the permeability of the glomerular filtration membrane. The glomerular filtration rate of an individual is dependent on its serum concentration. Because of its characteristics of continuous transcription and expression in animal cells with stable efficiency, Cys-C can be produced continuously and at a stable rate in the body. A previous report indicated that the sensitivity, specificity, and accuracy of determining abnormal glomerular filtration rate by Cys-C are better than Scr [12]. *α*1-MG is a low-molecular-weight protein that can be filtered through the glomeruli. Its characteristics of almost all reabsorption by the renal tubules are often used to indicate the reabsorption function of the renal tubules with good stability [13]. N-Acetyl-b-glucosaminidase is a cytolysosomal hydrolase that primarily reflects damage to the renal tubules. The increase of urinary NAG is mainly detected in renal tubular injury, which is a sensitive and specific indicator for renal tubular-interstitial disease. A recent study has indicated that urinary NAG can be used as a prognostic indicator of acute kidney injury [14]. mALB is a negatively charged mid-molecular-weight protein which can be detected from urine when the barrier function of the glomerular basement membrane is impaired. Thus, mALB is currently considered to be a more sensitive indicator of diabetic renal damage [15]. In this study, the blood Scr, blood Cys-C, urine *α*1-MG, urine mALB, and urine NAG were all significantly increased in the experimental rats 24 h after the injection of the contrast medium, and the increase was even higher at 72 h, indicating that the damage of kidneys is more serious with time. However, the serum Cys-C, blood Scr, urine *α*1-MG, urinary (NAG), and urine mALB in the RI group treated with rosuvastatin were significantly reduced as compared to the contrast control group (CM), indicating that short-term intervention with rosuvastatin had a positive effect on CIN protection.

The pathogenesis of CIN is the result of a combination of factors, among which the inflammatory response plays a key role in the occurrence of CIN [16]. In addition to the direct toxic effect of contrast media on renal tubular epithelium, oxygen-free radicals released by oxidative stress can also cause damage to renal tubular epithelial cells. The subsequent aggregation, adhesion, and activation of platelets promote the aggregation of inflammatory cells, which increases the synthesis and secretion of cytokines such as high-sensitivity C-reactive protein (Hs-CRP) from inflammatory cells. Inflammatory cytokines can cause inflammatory nuclear platelets to activate and form positive feedback. Numerous studies have shown that statins can reduce Hs-CRP levels [17]. In this study, the levels of Hs-CRP in the rat serum of the CM group were significantly increased over time, indicating that the rats had an inflammatory response after the injection of the contrast agent, which caused damage to renal function. All these confirm that the inflammatory response is an important cause of CIN. After injection of the contrast agent, the Hs-CRP level in the serum of rats in the RI group was significantly lower than that in the CM group, especially for the 72 h group. These results show that rosuvastatin can rapidly inhibit the inflammatory factor Hs-CRP, which may prevent CIN through its anti-inflammatory effect.

Clinical studies have shown that after injection of contrast agents in humans, a large amount of reactive oxygen species (ROS) are released, leading to acute kidney injury. SOD is an antioxidant enzyme that scavenges oxygen-free radicals and harmful substances. Studies have suggested that there is a negative correlation between SOD content and kidney damage [18]. Statins are known to be pleiotropic, including antioxidant properties, and they can prevent renal ischemia by stabilizing renal vascular endothelial cells and eliminating oxygen-free radicals, thereby preventing the occurrence of CIN [19]. This study revealed that SOD activity in rat kidney tissues decreased significantly over the time after injection of contrast media, indicating that oxidative stress is involved in the occurrence of contrast agent renal injury. In addition, the SOD activity level in rat kidneys was significantly higher in the RI group than in the CM group, indicating that rosuvastatin can enhance the antioxidant capacity of the rat body. The superoxide dismutase activity is enhanced, thereby improving the kidney damage in rats caused by contrast agents.

## 5. Conclusion

In summary, rosuvastatin can protect the renal function of rats with contrast-induced kidney injury to a certain extent, reduce the level of inflammation in rats, and enhance the ability of rats to resist oxidative stress. Specifically, following the intervention of rosuvastatin, various renal function indexes including Cys-C and urinary NAG were reduced. In addition, high-sensitivity C-reactive protein was significantly inhibited while SOD activity was significantly increased. Overall, rosuvastatin has a preventive and protective effect on contrast nephropathy, which may be mainly achieved through anti-inflammatory and antioxidative stress. Since this experiment failed to observe the protective effect of rosuvastatin on the contrast-induced renal injury model rats for a longer time, more in-depth studies are needed to confirm our findings and clarify the mechanism of rosuvastatin-mediated protection for renal injury induced by contrast agents in the future.

## Figures and Tables

**Table 1:** Comparison of renal tubular injury scores of experimental rats in different groups (*n* = 8).

Time (hr)	Renal tubular injury score		
	NC	CM	RI
24	0.26 ± 0.43	3.155 ± 0.61^*∗*^	2.01 ± 0.51^#^
72	0.28 ± 0.41	3.25 ± 0.66^*∗*^^▲^	1.99 ± 0.53^#▲^

^*∗*^*p* < 0.01 vs. the NS group; ^#^*p* < 0.01 vs. the CM group; ^▲^*p* < 0.05 vs. the 24 hr group.

**Table 2:** Changes of Scr and Cys-C in serum of experimental rats (*n* = 8).

Group	Scr (*μ*mol/L)		Cys-C (mg/L)	
	24 hr	72 hr	24 hr	72 hr
NC	29.16 ± 3.06	29.33 ± 2.93	0.034 ± 0.010	0.032 ± 0.009
CM	41.6 ± 3.36^*∗*^	53.5 ± 2.88^*∗*#^	0.108 ± 0.004^*∗*^	0.121 ± 0.021^*∗*#^
RI	35.4 ± 1.72^▲^	36.1 ± 1.68^▲^	0.064 ± 0.013^▲^	0.067 ± 0.008^▲^

^*∗*^*p* < 0.01 vs the NS group; ^▲^*p* < 0.01 vs the CM group; ^#^*p* < 0.05 vs the 24 hr group.

**Table 3:** Changes of *α*1-MG, mALB, and NAG in urine of experimental rats (*n* = 8).

Group	*α*1-MG (ng/mL)		mALB (ng/mL)		NAG (mIU/mL)	
	24 hr	72 hr	24 hr	72 hr	24 hr	72 hr
NC	126.93 ± 8.41	125.43 ± 7.92	46.93 ± 2.92	46.54 ± 2.34	17.51 ± 0.62	18.01 ± 0.93
CM	304.97 ± 6.52^*∗*^	331.16 ± 5.49^*∗*#^	65.98 ± 2.39^*∗*^	72.13 ± 2.39^*∗*#^	33.75 ± 1.91^*∗*^	38.74 ± 2.16^*∗*#^
RI	239.23 ± 6.76^▲^	240.36 ± 10.07^▲^	55.04 ± 4.02^▲^	55.765 ± 3.6^▲^	23.83 ± 1.52^▲^	23.21 ± 1.93^▲^

^*∗*^*p* < 0.01 vs. the NS group; ^▲^*p* < 0.05 vs. the CM group; ^#^*p* < 0.05 vs. the 24 hr group.

**Table 4:** Changes of Hs-CRP in serum of experimental rats (*n* = 8).

Time (hr)	Hs-CRP (*µ*g/L)		
	NC	CM	RI
24	435.33 ± 12.14	686.33 ± 13.20^*∗*^	532.13 ± 12.10^#^
72	446.45 ± 11.57	854.00 ± 10.50^*∗*^^▲^	480.38 ± 35.30^#▲^

^*∗*^*p* < 0.01 vs. the NS group; ^#^*p* < 0.01 vs. the CM group; ^▲^*p* < 0.05 vs. the 24 hr group.

**Table 5:** Changes of SOD activity in rat kidney tissues among experimental groups (*n* = 8).

Time (hr)	SOD (mgprot/mL)		
	NC	CM	RI
24	0.64 ± 0.01	0.38 ± 0.01^*∗*^	0.50 ± 0.01^#^
72	0.65 ± 0.01	0.29 ± 0.01^*∗*^^▲^	0.50 ± 0.01^#^

^*∗*^*p* < 0.01 vs. the NS group; ^#^*p* < 0.01vs. the CM group; ^▲^*p* < 0.05 vs. the 24 hr group.

## Data Availability

All the original experimental data will be available upon request to the first author.
